# The PCR-Based Diagnosis of Central Nervous System Tuberculosis: Up to Date

**DOI:** 10.1155/2012/831292

**Published:** 2012-05-13

**Authors:** Teruyuki Takahashi, Masato Tamura, Toshiaki Takasu

**Affiliations:** ^1^Department of Neurology, Nagaoka-Nishi Hospital Mitsugohya-machi, 371-1 Nagaoka City, Niigata, Japan; ^2^Division of Neurology, Department of Medicine, Nihon University School of Medicine, Tokyo, Japan

## Abstract

Central nervous system (CNS) tuberculosis, particularly tuberculous meningitis (TBM), is the severest form of *Mycobacterium tuberculosis *(*M.Tb*) infection, causing death or severe neurological defects in more than half of those affected, in spite of recent advancements in available anti-tuberculosis treatment. The definitive diagnosis of CNS tuberculosis depends upon the detection of *M.Tb* bacilli in the cerebrospinal fluid (CSF). At present, the diagnosis of CNS tuberculosis remains a complex issue because the most widely used conventional “gold standard” based on bacteriological detection methods, such as direct smear and culture identification, cannot rapidly detect *M.Tb* in CSF specimens with sufficient sensitivity in the acute phase of TBM. Recently, instead of the conventional “gold standard”, the various molecular-based methods including nucleic acid amplification (NAA) assay technique, particularly polymerase chain reaction (PCR) assay, has emerged as a promising new method for the diagnosis of CNS tuberculosis because of its rapidity, sensitivity and specificity. In addition, the innovation of nested PCR assay technique is worthy of note given its contribution to improve the diagnosis of CNS tuberculosis. In this review, an overview of recent progress of the NAA methods, mainly highlighting the PCR assay technique, was presented.

## 1. Introduction

Central nervous system (CNS) disease caused by *Mycobacterium tuberculosis *(*M.Tb*), particularly tuberculous meningitis (TBM), is uncommon and accounts for approximately 1% of all tuberculosis cases in the United States [1, 2]. CNS tuberculosis is the severest form of *M.Tb* infection, causing death or severe neurological defects in more than half of those affected, in spite of recent advancements in available antituberculosis treatment (ATT) [1–5]. In addition, owing to an increasing number of immunocompromised hosts caused by the prevalence of AIDS, increasing numbers of older people, the wider use of immunosuppressive agents, and other factors, TBM remains a serious clinical and social problem [1–5]. Owing to its relative rarity and the wide spectrum of its neurological symptoms, CNS tuberculosis remains a formidable diagnostic challenge [1–5]. In TBM, accurate and rapid diagnosis and early treatment for tuberculosis are the most important factors with regard to the prognosis and the prevention of long-term neurological sequelae [1–5]. However, the conventional “gold standard” based on bacteriological detection methods of *M.Tb*, including the direct smear examination for acid-fast bacilli (AFB) and culture identification, is inadequate for early diagnosis, owing to the poor sensitivity or the long time required (4–8 weeks) for cultures [1–5].

 Recently, the detection of *M.Tb* DNA in the cerebrospinal fluid (CSF) through the use of various molecular-based methods, including nucleic acid amplification (NAA) assay technique, particularly polymerase chain reaction (PCR) assay, has emerged as a promising new method for the diagnosis of CNS tuberculosis because of its rapidity, sensitivity, and specificity [2–42]. Many investigators have reported on the usefulness of PCR assay for the detection of *M.Tb *DNA in CSF, although the sensitivity of PCR assay has significant discrepancies (65–83%) between each type of measuring methods and different laboratories [2–42]. Moreover, nested PCR assay has been reported as a prominent method for detecting *M.Tb* DNA in CSF [3, 4, 17, 19, 24–27, 32]. This new method drastically increases the sensitivity and specificity of DNA amplification compared with conventional single-step PCR [3, 4, 17, 19, 24–27, 32]. However, the nested PCR assay using CSF samples has yet to be widely used in TBM diagnosis owing to its laborious and time-consuming procedure, which carries a high risk of cross-contamination [3, 4, 17, 19, 24–27, 32]. Currently, real-time PCR assay is applied in routine diagnostic laboratory testing [33, 38, 40–42]. In addition to conventional qualitative analysis, real-time PCR assay makes it possible to perform accurate quantitative analyses with a high degree of reproducibility [33, 38, 40–42].

 In this paper, the authors highlight the recent advancement of NAA assay techniques, in particular PCR assay and provide an overview of the current issues and evolution of diagnosis and clinical aspects of CNS tuberculosis.

## 2. The Global Epidemiologic Burden of Tuberculosis

In 2007, the World Health Organization (WHO) estimated that 9.27 million new cases (139/100,000 population) of active tuberculosis occur annually, resulting in an estimated 1.6 million deaths per year [1, 2]. Tuberculosis remains a worldwide burden, with a large majority of new active tuberculosis cases occurring in underdeveloped and developing countries [1, 2]. In fact, India, China, Indonesia, Nigeria, and South Africa rank first to fifth in the total number of incident cases of tuberculosis [1, 2]. In 80% of new tuberculosis cases, social and demographic factors such as poverty, overcrowding, malnutrition, and a compromised immune system play a major role in the worldwide epidemic, while the remaining 20% of tuberculosis cases are associated with HIV in sub-Saharan Africa [1, 2]. Among the 9.27 million new tuberculosis cases in 2007, an estimated 1.37 million (14.8%) were HIV positive [1, 2].

 CNS infection is one of the severest forms of tuberculosis [1–5]. In a large-scale epidemiological study of extrapulmonary tuberculosis in the United States, CNS involvement was noted in 5 to 10% of extrapulmonary tuberculosis cases, with more recent CDC data in 2010 indicating that 5.5% of extrapulmonary cases involve CNS tuberculosis (=1.2% of total tuberculosis cases) [43–46]. In the largest prospective epidemiological study of CNS tuberculosis, the incidence of developing CNS tuberculosis was approximately 1.0% among 82,764 tuberculosis cases from 1970 to 2001 in a Canadian cohort [1, 2, 43]. However, despite an overall decrease in the total number of tuberculosis cases in advanced nations such as the United States, a gradual and continuous increase in the proportion of extrapulmonary tuberculosis cases has been reported [1, 2, 43–46]. This increase has been mainly attributed to the recent increase of immunocompromised patients and the HIV/AIDS epidemic [1, 2, 43–46]. In addition, although the overall population-based mortality rate from tuberculosis is low and decreasing, several studies have shown that mortality rates are substantially higher in patients with several forms of extrapulmonary tuberculosis, including CNS tuberculosis or TBM and disseminated disease [1, 2, 43–46].

 Several risk factors for CNS tuberculosis have been identified. Both age (children > adults) and HIV-coinfected patients are at high risk for developing CNS tuberculosis [1, 2, 44]. Other risk factors include malnutrition, recent measles in children, alcoholism, malignancies, the use of immunosuppressive agents in adults, and disease prevalence in the community [1, 2]. Several studies in developed countries have also identified that foreign-born individuals (individuals born outside of developed countries) are overrepresented among CNS tuberculosis cases [1, 2, 44–46].

## 3. Current Issues for Diagnosis in CNS Tuberculosis

At present, the diagnosis of CNS tuberculosis remains a complex issue because the most widely used conventional bacteriological detection methods, such as direct smear for AFB and culture for *M.Tb*, cannot rapidly detect *M.Tb *in CSF specimens with sufficient sensitivity in the acute phase of TBM [2–42]. Rapid and accurate diagnosis in the acute phase of CNS tuberculosis and early starting ATT are the most important factors with regard to the prognosis and the prevention of long-term neurological sequelae [2–5]. The poor sensitivity and often delayed results from the conventional “gold standard” based on microbiological techniques in the traditional TBM diagnosis underscore the need for a more sensitive, rapid, and accurate diagnostic method in clinical practice [2–5]. Several molecular-based methods, often drawn from successful techniques used for the diagnosis of tuberculosis in respiratory specimens, have been evaluated for their applicability in the diagnosis of CNS tuberculosis. These techniques include commercially available NAA methods and other PCR-based methods [2–42]. In addition, the use of neuroradiographic techniques such as magnetic resonance imaging (MRI) has prominently improved the diagnostic accuracy of TBM and tuberculomas [2, 47–49]. Recently, the role of neuroradiographic techniques in the evaluation of CNS tuberculosis has been reviewed in various reports [1–5, 47–49]. Commonly identified neuroradiological features of TBM include basal meningeal enhancement, hydrocephalus, and infarctions in the supratentorial brain parenchyma and brain stem [2, 47–49]. Moreover, tuberculomas are generally defined as low- or high-intensity, round or lobulated masses with irregular walls and show homogeneous or ring enhancement after the administration of contrast [2, 47–49]. They occur as solitary or multiple nodules and are typically found in the frontal and parietal lobes [2, 47–49]. However, the differential diagnosis of tuberculomas and other intracranial focal massive lesions such as fungal granulomas is difficult when using only neuroradiographic techniques [2]. Therefore, the combination of molecular-based techniques and neuroradiographic techniques is regarded as a promising and powerful diagnostic tool for TBM and tuberculomas, instead of neuroradiographic techniques with or without classical bacteriological detection methods [2].

## 4. Recent Advancement of PCR Assay Technique

The NAA methods for *M.Tb* are diagnostic techniques to demonstrate the presence of tubercle bacilli by the extraction and amplification of DNA or RNA of *M.Tb* from clinical specimens such as sputum or CSF. The representative DNA amplification method is the PCR assay technique. In this section, an overview of the principles of PCR assay techniques is presented, mainly with regard to their recent advancement and evolution.

### 4.1. The Basic Principle of PCR Assay

**Figure 1:**
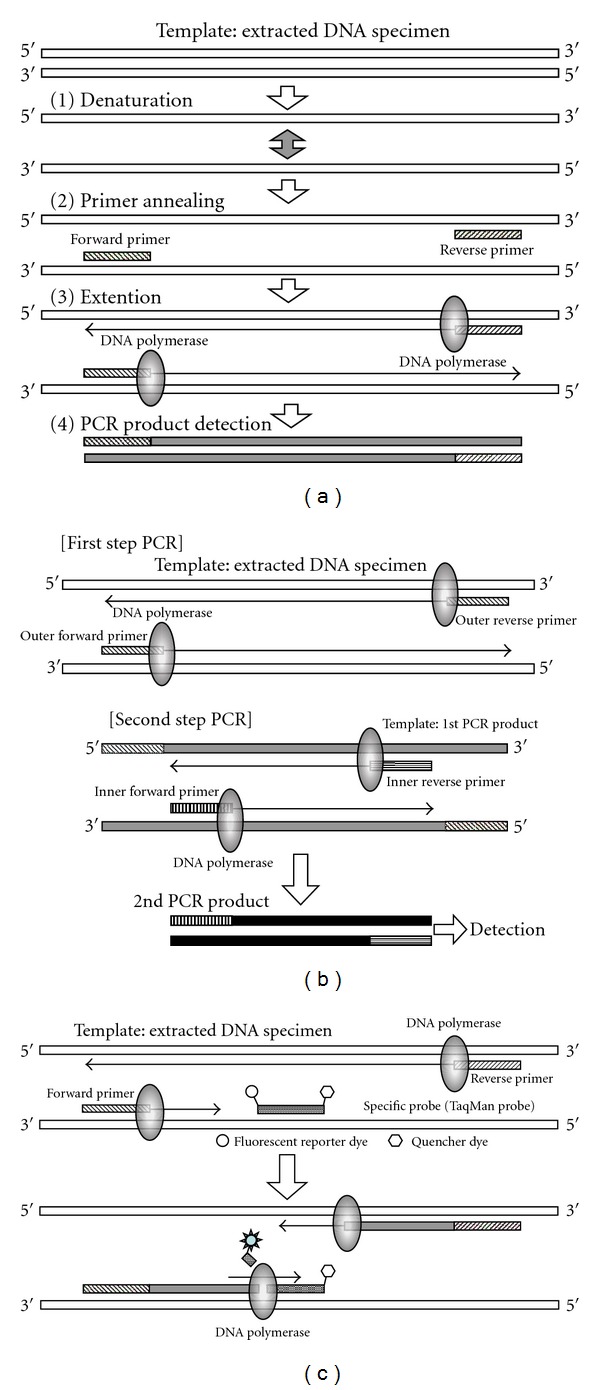
Schemata of the principles of polymerase chain reaction (PCR) assay techniques. (a) A schema indicating the basic principle of PCR assay. (b) A schema indicating the principle of nested PCR assay. (c) A schema indicating the principle of real-time (TapMan) PCR assay.

A schema indicating the basic principle of the PCR assay is shown in Figure 1(a). Fundamentally, the PCR assay technique depends on “thermal cycling,” consisting of cycles with repeated heating and cooling for the reactions of DNA denaturation and enzymatic replication of DNA. Short DNA fragments called “primers” containing sequences complementary to the target region along with a DNA polymerase are key components to enable sequence-specific DNA amplification. The thermal cycling procedure involves a first step for physical separation of the two strands of DNA double helix at a high temperature (94–98°C for 20–30 seconds); this is called the DNA denaturation step. At a lower temperature (50–65°C for 20–40 seconds), the primers that are complementary to the target DNA region anneal to each separated single-stranded DNA as the template; this is called the annealing step. The specificity of PCR mainly results from both the primer sequence setting that closely matches the template sequence and the annealing temperature setting depending on the length of primers. The DNA polymerase binds to the primer-template hybrid and begins DNA synthesis. The DNA polymerase enzymatically assembles and synthesizes a new DNA strand complementary to the DNA template in the 5′ to 3′ direction; this is called the extension/elongation step. In general, almost all PCR applications employ a heat-stable DNA polymerase, such as Taq polymerase, an enzyme originally isolated from the bacterium *Thermus aquaticus*. As a result, it is possible to repeat serially the thermal cycling procedure, consisting of alternate heating and cooling steps. As the thermal cycling procedure progresses, the synthesized DNA fragment is itself used as a template for replication, setting in motion a “chain reaction” in which the DNA template is exponentially amplified.

Through the use of agarose gel electrophoresis, the amplified DNA fragments can be separated by their lengths (molecular weights). The agarose gel is then treated with a solution containing ethidium bromide (EtBr), which is the most commonly used dye for visualizing DNA bands in agarose gel electrophoresis. Because EtBr fluoresces under UV light when intercalated into the major groove of DNA, through EtBr treatment, the amplified target DNA fragment can be visualized and detected as a distinctive band.

### 4.2. The Principle of Nested PCR Assay

A schema indicating the principle of nested PCR assay is shown in Figure 1(b).

 Nested PCR assay is a modified version of the PCR technique intended to increase the amplification efficiency markedly and to reduce the level of nonspecific PCR products due to the amplification of untargeted primer binding sites. Although the specificity of the standard single-step PCR depends on the primers′ complementarity to the target DNA sequence, a commonly occurring problem is that primers bind to other similar regions of the DNA, giving untargeted PCR products such as primer dimers, hairpins, and alternative primer target sequences. Nested PCR assay requires two sets of primer pairs, used in two successive steps of the PCR procedure. In particular, a second set of primer pairs is prepared to amplify a secondary target within the first-step PCR product; this is the source of the term “nested”. The first set of primers amplifies a fragment similarly to the standard single-step PCR. However, the second set of primers binds inside the first-step PCR product to allow amplification of the second-step PCR product, which is shorter than the first one. The advantage of the nested PCR assay is that, if an untargeted or nonspecific PCR product is amplified, the probability is quite small that the region would be amplified in the second-step PCR by the second set of primers. Thus, nested PCR assay is an excellent technique for obtaining a sufficient amount of target DNA through a two-step amplification procedure, and for prominently improving the specificity to the target sequence by reducing the amplification of nonspecific products.

### 4.3. The Principle of Real-Time Quantitative PCR Assay

Real-time PCR assay is a variation of the PCR technique intended to amplify and simultaneously quantify a targeted DNA molecule; it enables both detection and quantification. Although the basic procedure of real-time PCR follows the general principle of classical PCR, its key feature is that the amplified DNA fragment is detected as the reaction progresses in “real time.” This is a novel and revolutionary approach compared with conventional standard PCR, where the PCR product is detected at the end of the reaction procedure. Two common methods for detection of products in real-time PCR are as follows: (1) nonspecific fluorescent dyes such as SYBR Green that intercalate with any double-stranded DNA and (2) sequence-specific oligonucleotide probes such as TaqMan probe that are labeled with a fluorescent reporter dye, which permits detection only after hybridization of the probe with its complementary target sequence. The former (SYBR Green) will bind to all double-stranded DNA PCR products including nonspecific PCR products (such as primer dimer). This is a potential disadvantage as it could obstruct accurate quantification of the intended target DNA fragment.

 In contrast, fluorescent reporter probes such as TaqMan probe detect only the DNA fragment containing the complementary probe sequence; therefore, use of such reporter probes significantly increases the specificity and enables accurate quantification, even in the presence of nonspecific amplified fragments. A schema indicating the principle of real-time quantitative PCR assay based on a fluorescent reporter probe (TaqMan PCR) is shown in Figure 1(c). The sequence-specific oligonucleotide probe is labeled with a fluorescent reporter dye, such as FAM or VIC, at the 5′-end and conjugated with a quencher dye, such as TAMRA, at the 3′end. The close proximity between a fluorescent reporter dye and a quencher dye prevents emission of its fluorescence. As the reaction is initiated, both probe and primers anneal to the target DNA sequence during the annealing step of PCR. As the PCR procedure progresses, breakdown of the probe by the 5′ to 3′ exonuclease activity of Taq polymerase separates the reporter from the quencher and thus allows unquenched emission of fluorescence of the reporter dye, which can be detected after excitation with a laser. The strength of fluorescence increases exponentially because fluorescent reporter dyes separate from quencher dyes in a manner corresponding to the progress of PCR cycles, and its geometric increase is used to determine the threshold cycle (*Ct*) value in order to calculate the amplification rate in each reaction. As a result, the primary amount of DNA can be quantified accurately.

## 5. Commercially Available NAA Methods for *M.Tb*

Currently, two commercially available NAA methods for the direct detection of *M.Tb* complex have been approved by the United States Food and Drug Administration (FDA), as follows: Roche Amplicor *Mycobacterium tuberculosis* Test (Roche Diagnostic Systems, Inc., Indianapolis, IN, USA) and Gene-Probe Amplified *Mycobacterium tuberculosis* Direct (MTD) Test (Gene-Probe, Inc., San Diego, CA, USA) [2, 6–12]. Both tests use the 16S ribosomal (r) RNA gene of *M.Tb* (GenBank accession no. NC_000962.2 (1471846–1473382)) as the target sequence for amplification [2, 6–12]. The 16S rRNA gene represents a stable property of microorganisms and is widely used as the target for identifying mycobacterial species [2, 6–12].

 Roche Amplicor Test involves the conventional standard PCR-based method. In this test kit, the DNA fragment is amplified and detected by the primer pair and probe that are specific for the 16S rRNA gene of *M.Tb* [2, 6, 8, 10]. In addition, Roche Cobas TaqMan MTB Test is the improved successor to the Amplicor Test and adopts a real-time (TaqMan) PCR assay technique. Meanwhile, Gen-Probe MTD Test is the isothermal amplification method for RNA [2, 7, 9–12]. In this test kit, the 16S rRNA of *M.Tb* is directly amplified by the coupling of reverse transcriptase and RNA polymerase under a constant temperature (43°C), and detected by hybridization using specific oligo-RNA probe [2, 7, 9–12]. At present, Amplicor is approved by the FDA for testing of AFB smear-positive respiratory specimens only [2, 6–12]. Meanwhile, MTD is approved for testing of respiratory specimens, regardless of the result of smear for AFB [2, 6–12]. Several studies have reported excellent results for both tests (sensitivity and specificity levels of more than 95%) in AFB smear-positive respiratory specimens, but reduced sensitivity (60 to 85%) when applied for AFB smear-negative respiratory specimens [2, 6–12]. Neither test is approved by the FDA for testing of CSF specimens [2, 6–12].

## 6. Clinical Application of PCR Assay Technique for the Diagnosis of CNS Tuberculosis

Table 1 summarizes the performance of PCR-based assays for the diagnosis of CNS tuberculosis [2, 6–42]. The challenges of applying NAA assay techniques to the rapid diagnosis of *M.Tb* in the CSF specimens stem largely from the small number of bacilli typically present in TBM and the presence of amplification inhibitors in the CSF [2–42]. In actual clinical practice, the sensitivity and specificity of PCR-based assay methods are the most serious issues in the diagnosis of CNS tuberculosis. In order to improve both the sensitivity and the specificity of PCR assay, the efficient extraction and purification of DNA from a small number of *M.Tb* bacilli in the CSF specimens and the setting of primers to amplify *M.Tb* DNA as the template specifically and efficiently are the most important factors [2–42]. Therefore, many researchers have worked intensively on these issues [2–42]. In the 1990s, a number of improved extraction and purification methods of *M.Tb* DNA from CSF specimens were reported by Shankar et al. [14], Kaneko et al. [13], and Lin et al. [20], According to these studies, the CSF specimen was treated with cytolysis buffer containing proteinase-K and sodium dodecyl sulfate (SDS) as a surface-active agent, and then *M.Tb* DNA was extracted and purified using phenol-chloroform and ethanol precipitation from the treated CSF specimen [13, 14, 20]. In order to extract a small amount of *M.Tb* DNA from a CSF specimen more efficiently, the authors used a high-molecular-weight carrier, Ethachinmate (Nippon Gene, Tokyo, Japan), as a coprecipitating agent for the nucleotides together with the previously reported phenol-chloroform extraction and ethanol precipitation [3, 4, 32, 33, 38, 40, 41]. However, in recent studies, the conventional phenol-chloroform extraction and ethanol precipitation have tended to be regarded as inadequate for routine use in clinical examinations because of their laborious and time-consuming procedures [25–31, 34–37]. Generally, commercially available column extraction kits, such as the QIAmp Blood Kit and QIAmp DNA Mini Kit (Qiagen Inc., Valencia, CA, USA), and the Cobas Amplicor respiratory specimen preparation kit (Roche Diagnostic Systems, Inc., Indianapolis, IN, USA), have been widely used for DNA extraction from various samples in previous studies [6–12, 25–31, 34–37]. However, in the current study, it was impossible to extract *M.Tb* DNA from CSF specimens sufficiently using commercial column extraction kits; therefore, these popular kits may be inadequate for extracting a small amount of *M.Tb* DNA from CSF specimens [3, 4, 32, 33, 38, 40, 41].

 Currently, the four major *M.Tb* DNA-specific sequences, including the regions of IS6110 insertion sequence (Rv3475: GenBank accession no. NC_000962.2 (3891051–3892091)), 65-kDa heat shock protein antigen (Rv0251c: NC_000962.2 (302173–302652)), 16S rRNA gene and MPT64 (NC_000962.2 (2223343–2224029)) are evaluated by NAA assays (Table 1) [6–42]. Of these four sequence regions, the IS6110 insertion sequence, which is a repetitive element exclusively found in the genome of *M.Tb* complex, has been most widely used as the target sequence with superior amplification efficiency in many studies (Table 1) [15, 16, 18, 19, 21, 22, 24, 25, 28, 34, 36, 37, 39]. In these previous studies, PCR assays targeting the IS6110 insertion sequence revealed relatively good results (an overall sensitivity of 70–98% and specificity of 80–100%) for TBM diagnosis (Table 1). In addition, next to the IS6110 insertion sequence, the 16S rRNA gene has been frequently used for NAA assays, and it is the target sequence of two commercially available *M.Tb* detection methods, namely, the Roche Amplicor Test and the Gen-Probe MTD Test, which have been approved by the FDA for testing of respiratory specimens, as described above [2, 6–12]. At present, no commercially available NAA assay methods have been approved for testing of CSF, but several studies have evaluated their performance in TBM cases (Table 1) [2, 6–12]. A recent meta-analysis concerning the accuracy of commercially available NAA assay methods for TBM diagnosis revealed an overall sensitivity of 56% and specificity of 98% [10]. On the basis of these results, the commercially available NAA assay methods are evaluated as follows: they may play a role in confirming TBM, but because of low sensitivity, they are not ideal for ruling out TBM [2, 10]. As the major reason for the insensitive performance of these two commercially available NAA assay methods for TBM diagnosis, it is considered that, since they have been approved for testing of respiratory specimens containing relatively large numbers of *M.Tb* bacilli, they are inadequate for detecting a small amount of *M.Tb* DNA from CSF specimens. Recently, in several studies, Gen-Probe MTD Tests modified to improve the performance for detecting *M.Tb* DNA in CSF specimens have been reported [7, 9, 11, 12]. However, the use of these modified techniques was limited to a single laboratory, and they have not been widely used [7, 9, 11, 12]. Meanwhile, MPT64 is the gene sequence encoding the MPB64 protein that is specific for *M.Tb* complex and exists at only one site on the *M.Tb* genome. Therefore MPT64 is regarded as the most specific sequence for PCR assays and has been used as the target sequence for PCR in many studies (Table 1) [3, 4, 13, 14, 16, 17, 20, 23, 26, 27, 32, 33, 38, 40, 41]. Lee et al. [16] compared three sequence regions (IS6110, 65 kDa antigen, and MPT64) and reported that MPT64 was the most specific and sensitive sequence for the amplification of *M.Tb* DNA by PCR.

 Beyond the commercially available NAA methods, the application of PCR-based methods to amplify *M.Tb* DNA has received a lot of attention clinically (Table 1) [13–42]. As described above, in order to improve the performance of the PCR-based assays for the diagnosis of CNS tuberculosis, various ideas and modifications have already been tried [2, 6–42]. Recently, as a revolutionary assay technique that conveys drastically increased sensitivity and specificity compared with the conventional standard PCR assay, nested PCR assay has been innovated for the diagnosis of CNS tuberculosis [3, 4, 17, 19, 24–27, 32]. Liu et al. [17] reported that nested PCR assay targeting MPT64 was performed for CSF specimens collected from 21 patients with clinically suspected TBM and enabled diagnosis of TBM with a sensitivity of 90% and a specificity of 100% within 24 hours. In addition, they reported that the nested PCR assay had approximately 1000 times higher sensitivity than the conventional single-step PCR assay [17]. The authors performed the nested PCR assay targeting MPT64 for the CSF specimens collected from 9 patients with clinically highly suspected TBM [32]. In our study, both the sensitivity and the specificity of nested PCR assay were 100%, but the sensitivity of the conventional single-step PCR assay and culture for *M.Tb* was only 22.2% [32]. Moreover, the minimum detection sensitivity of our nested PCR assay technique was examined. This technique enabled detection at a level as low as 1–10 copies/2 *μ*L of purified *M.Tb* DNA and had 1000 to 10,000 times higher sensitivity than the conventional single-step PCR assay [32]. Concerning the relationship between the PCR assay results and the responses for ATT, Lin et al. [20] examined this relationship by conventional single-step PCR assay and Scarpellini et al. [19] examined it by nested PCR assay. In particular, Scarpellini et al. [19] performed diachronic study by nested PCR assay targeting IS6110 for serial CSF specimens collected from 7 TBM patients during the clinical treatment course. In their diachronic study, the nested PCR assay results were converted from positive to negative in 4 TBM patients whose clinical conditions recovered during ATT [19]. In contrast, the nested PCR assay results remained positive throughout the clinical course in 3 patients who demonstrated no ATT response and died [19]. Therefore, Scarpellini et al. concluded that the nested PCR assay was useful for assessing the clinical treatment course of TBM [19]. Similarly, in our study, 11 out of 27 serial CSF specimens that were available from the 7 patients with highly suspected TBM and collected during the clinical treatment course of ATT revealed positive results (40.7%) of the nested PCR assay targeting MPT64 [32]. Moreover, our nested PCR assay results were converted from positive to negative in the 6 patients whose clinical conditions recovered during the ATT [32]. In contrast, the conventional methods including single-step PCR assay and culture for *M.Tb* all revealed negative results for a series of 27 CSF specimens during the clinical treatment course of 7 patients [32]. The nested PCR assay is a useful assay technique with superior sensitivity and specificity. Because of the demonstration of the capacity of the nested PCR assay in the diagnosis of difficult cases in which other conventional assay methods cannot detect *M.Tb*, the authors speculated that, if this assay technique was widely and appropriately used within clinical practice, it would be a powerful tool for the rapid and accurate diagnosis of CNS tuberculosis.

 Currently, although the wide use of nested PCR assay in clinical practice is expected, regrettably, it has rarely been performed for TBM diagnosis [3, 4, 17, 19, 24–27, 32]. The main argument against the use of nested PCR assay is that, because of its complicated, laborious, and time-consuming procedures, it is an inadequate assay technique for routine use in clinical examination [3, 4, 17, 19, 24–27, 32]. In addition, owing to its markedly increased sensitivity and the requirement for an additional amplification step, in general, it is considered that cross-contamination can easily occur through the nested PCR assay procedure [3, 4, 17, 19, 24–27, 32]. However, the possibility of cross-contamination can be minimized by good laboratory practice. Certainly, the nested PCR assay may be inadequate for routine use in clinical examinations dealing with many sample specimens such as screening examinations. However, even if any other “simple” assay methods are used for TBM diagnosis, a negative result cannot exclude the possibility of *M.Tb* infection, and clinical judgment in TBM diagnosis remains a serious problem with the requirement of an appropriate clinical examination [2]. In actual clinical practice, the diagnosis of CNS tuberculosis requires not a screening examination but rather a definitive examination. Therefore, the nested PCR assay should become a prominent and useful assay technique if used on well-defined and appropriate clinical specimens collected from “highly suspected” TBM patients. Finally, a remaining challenge of using PCR-based assay methods in the diagnosis of CNS tuberculosis is the requirement for an appropriate laboratory infrastructure to perform these more sophisticated assay techniques; it is a fact that the infrastructure is often lacking in areas where TBM is highly endemic. This is a crucial issue that should be solved as soon as possible in the diagnosis of CNS tuberculosis.

## 7. Development of Novel Diagnostic Assay Technique Based on PCR for CNS Tuberculosis

**Figure 2:**
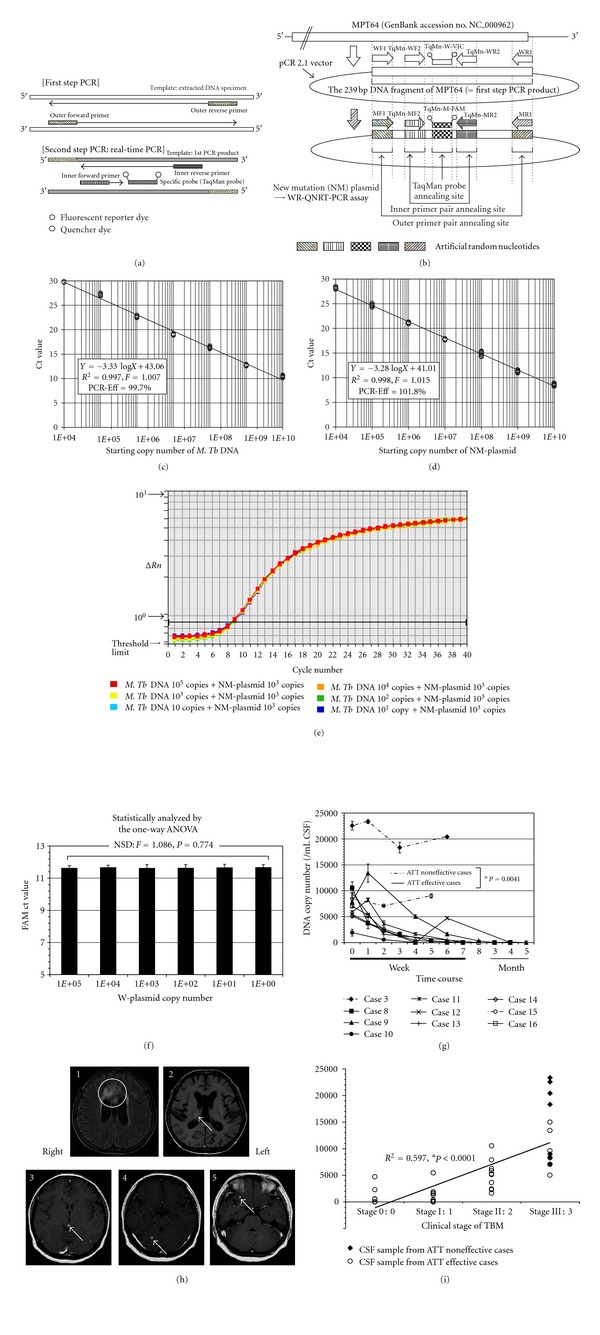
The principle of wide-range (WR) quantitative nested real-time (QNRT) PCR assay and its results. (a) A schema indicating the principle of WR-QNRT-PCR assay. (b) A schema of wild (W) and new mutation (NM) plasmids NM-plasmid was developed for use as a new internal control. (c) The specific standard curve to detect *Mycobacterium tuberculosis *(*M.Tb*) DNA or W-plasmid quantitatively. (d) The specific standard curve to detect the NM-plasmid as a new internal control quantitatively. (e) Amplification curves for 10^3^ copies of NM-plasmids as a new internal control. (f) One-way ANOVA against *Ct* values for 10^3^ copies of NM-plasmid. (g) The progress of *M.Tb* DNA copy numbers calculated by the WR-QNRT-PCR assay during clinical time course in 10 suspected tuberculous meningitis (TBM) patients (cases 3 and 8–16). Statistical comparison between the cases in which anti-tuberculosis treatment (ATT) was effective (cases 8–14 and 16) and those in which it was not effective (cases 3 and 15) was carried out by repeated-measure ANOVA. (h) Cranial MRI findings for cases 11 and 12 on admission. 1, 2: Cranial MRI findings for case 11. 1: FLAIR image (TR 9000/TE 110). High-intensity lesions of cerebral infarction (circle), which were probably caused by tuberculous vasculitis, are noted on both sides of the frontal lobe. 2: T1-WI (TR 500/TE 17). A cranial tuberculoma was noted in the right thalamus. 3, 4, 5: Cranial MRI findings (Gd T1-WI: TR 400/TE 15) for case 12. Multiple cranial tuberculomas with marked Gd enhancement (arrows) were noted (3: midbrain, 4: right cerebellum, 5: right temporal lobe). (i) Result of simple regression analysis between *M.Tb* DNA copy number (*y* axis) and clinical stage of TBM (*x* axis).

Recently, the authors developed an internally controlled novel “wide-range (WR)” quantitative nested real-time (QNRT) PCR assay for *M.Tb* DNA, in order to rapidly diagnose CNS tuberculosis [32, 33, 38, 40, 41]. This technique combines the high sensitivity of nested PCR with the accurate quantification of real-time (TaqMan) PCR (Figure 2(a)) [40].

In WR-QNRT-PCR assay, two types of original plasmid, “wild” (W) and “new mutation” (NM) plasmids, were prepared for quantitative detection of *M.Tb* DNA [40, 41]. W-plasmid, which was inserted a 239-base-pair (bp) DNA fragment of the MPT64 gene into pCR 2.1 vector (Invitrogen Corp., San Diego, CA, USA), was constructed for use as standard template (Figure 2(b)) [40]. NM-plasmid was developed on the basis of the W-plasmid for use as a new internal control “calibrator” in WR-QNRT-PCR assay (Figure 2(b)) [40]. In NM-plasmid, a total of five regions, where two pairs of (outer and inner) forward and reverse primers and TaqMan probe annealed, were replaced with artificial random nucleotides (Figure 2(b)) [40]. The sequences of the artificial random nucleotides were set to have the same nucleotide composition as MPT64 of wild *M.Tb* [40, 41]. For use in WR-QNRT-PCR assay, four pairs of specific primers and two types of specific TaqMan probes were prepared. The sequences and positions of these primers and probes are shown in Table 2 and Figure 2(b) [40, 41]. The two pairs of forward and reverse primers (outer primer pair WF1 and WR1 for use at the first step and inner primer pair TqMn-WF2 and TqMn-WR2 for use at the second step) and TaqMan probe (TqMn-W-VIC) were specific for MRT64 of wild *M.Tb* or W-plasmid. In contrast, the two pairs of forward and reverse primers (outer primer pair MF1 and MR1 and inner primer pair TqMn-MF2 and TqMn-MR2) and TaqMan probe (TqMn-M-FAM) were specific for the artificial random nucleotides in NM-plasmid for use as a new internal control “calibrator.” These primers and probes were set to have the same nucleotide composition but a different and random sequence (Table 2) [40, 41]. Therefore, the annealing efficiencies of these primers and probes to wild MPT64 or NM-plasmid as a template can be regarded as the same.

 WR-QNRT-PCR assay consists of two consecutive PCR amplification steps, which are conventional PCR at the first step and real-time (TaqMan) PCR at the second step (Figure 2(a)) [33, 38, 40, 41]. In this assay, the entire procedure including extraction, amplification, and detection for both *M.Tb* DNA and NM-plasmid as a new internal control is performed simultaneously under the same assay conditions by using four pairs of primers and two probes that have equivalent annealing efficiency to the respective templates. Therefore, the initial copy number of *M.Tb* DNA in CSF samples can be calculated by the amplification ratio against the new internal control (NM plasmid) as a “calibrator,” using the following equation; X : W = C : M *∴*X = W × C/M. (X, the initial copy number of* M.Tb* DNA per 1 mL of CSF sample; C, the initial copy number of the new internal control (=“calibrator” 10^3^ copies of NM-plasmid); W and M, the copy numbers of *M.Tb* DNA and NM-plasmid, respectively, after passing through extraction and PCR amplification procedures.) [40, 41] In *M.Tb*, it is universally acceptable that a single copy of MPT64 gene represents one bacterial cell. Therefore, it can be considered that the copy numbers calculated by WR-QNRT-PCR assay correspond to the bacterial cell numbers of *M.Tb* in CSF samples.

 For WR-QNRT-PCR assay, two specific standard curves for quantitative detection of *M.Tb* DNA and NM-plasmid as a new internal control are needed [40, 41]. The precision of the standard curve is the principal factor for quantitative detection in real-time (TaqMan) PCR assay [40, 41]. The two specific standard curves are shown in Figures 2(c) and 2(d) [40, 41]. In simple regression analysis, both of these two standard curves demonstrated a significant linear relationship (*R* ^2^ > 0.99) between the threshold cycle (*Ct*) values (*y* axis) and log of the starting copy numbers for each standard template (*x* axis). In both standard curves, no significant differences were found among the plots in repeated experiments (*n* = 10; *F* = 1.007, *P* = 0.65 and *F* = 1.015, *P* = 0.53) by two-way ANOVA. The PCR-efficiency (Eff) of real-time PCR can be calculated by the slope of the standard curve, in the following equation: PCR − Eff = 10^(−1/slope)^ − 1. The PCR-Eff calculated by this equation based on the slopes (−3.33 or −3.28) of two standard curves was 99.7 or 101.8%, respectively [40, 41]. These results indicated that the efficiencies of amplification and detection for both *M.Tb* DNA and the new internal control were almost equivalent in the WR-QNRT-PCR assay.

 When a value of 10^3^ copies of NM-plasmid was set as the optimal copy number of new internal control, the amplification curves of NM-plasmids revealed extremely uniform patterns in all starting copy numbers (1–10^5^) of W-plasmids as a mimic of *M.Tb *DNA (Figure 2(e)) [40]. In addition, the *Ct* values for 10^3^ copies of the NM-plasmid also revealed statistically significant uniform variance between all starting copy numbers of W-plasmids (*F* = 1.086, *P* = 0.774) by one-way ANOVA (Figure 2(f)) [40]. These results indicated that there was no interference between *M.Tb *DNA and the new internal control in the entire PCR amplification procedures. Therefore, NM-plasmid could be regarded as appropriate for use as a new internal control “calibrator” in WR-QNRT-PSR assay. Owing to the development of NM-plasmid, the WR-QNRT-PCR assay enabled statistically significant stable and accurate quantitative detection of *M.Tb *DNA with a wide detection range (1–10^5^ copies) [40, 41].

 The authors examined the clinical usefulness of the WR-QNRT-PCR assay for rapid and accurate diagnosis and assessment of the clinical course of CNS tuberculosis [41]. Twenty-four patients with clinically suspected TBM and 29 non-TBM control patients were collected between 1998 and 2005 [41]. A total of 67 CSF samples were collected from these 24 patients. Of 67 CSF samples, 43 were available serially from the 10 patients (cases 3 and 8–16) who had followups of more than 2 weeks [41].  Table 3 summarizes the clinical features of the 24 suspected TBM patients upon admission (before ATT) [41]. All 24 patients met the (A) clinical criteria and (B) supporting evidence for TBM (shown in Table 3) and were classified as 8 “confirmed” cases (cases 1 to 8) (CSF culture positive for *M.Tb*) and 16 “highly probable” cases (cases 9 to 24) (meeting all the clinical criteria and with three positive results for supporting evidence, but having no bacterial isolation). In clinical applications, the WR-QNRT-PCR assay revealed sufficiently high sensitivity (95.8%) and specificity (100%) for 24 clinically suspected TBM patients [41]. The measured copy numbers of *M.Tb* DNA (per 1 mL of CSF) are shown in Table 3 [41]. In conditional logistic regression analysis, a copy number of *M.Tb* DNA (per 1 mL CSF) >8000 was an independent risk factor for poor prognosis of TBM (=death) (OR = 16.142, 95%CI = 1.191–218.79, *P = 0.0365) [41]. In the diachronic study, the copy numbers of *M.Tb* DNA demonstrated significant alterations during the clinical treatment course in 10 suspected TBM patients, in these 10 patients including 2 “confirmed” cases (cases 3 and 8) and 8 “highly probable” cases (cases 9–16) [41]. The classical cultures for *M.Tb* revealed positive results in only three out of 43 serial CSF samples collected during the clinical treatment course in cases  3 and 8. In contrast, the quantitative detection of *M.Tb* DNA was possible in 25 CSF samples (58.1%) in the WR-QNRT-PCR assay [41]. In addition, the copy numbers of *M.Tb* DNA decreased gradually to below the detection limit of the WR-QNRT-PCR assay in the 8 patients (cases  8–14 and 16) for whom ATT was effective and who demonstrated improvement in both clinical stages and routine CSF findings during clinical treatment course (Figure 2(g)) [41]. However, in cases 3 and 15, in whom ATT was not effective and who died due to aggravation of TBM, the copy numbers were continually high throughout the clinical course (Figure 2(g)) [41]. The trend in the alterations of *M.Tb* DNA copy numbers during clinical treatment course demonstrated a significant difference (*P = 0.0041) between the ATT effective cases (cases  8–14 and 16) and the ATT noneffective cases (cases 3 and 8) by repeated-measure ANOVA (Figure 2(g)) [41]. In cases 11 and 12, initial cranial MRI on admission demonstrated multiple intracranial focal masses that were regarded as typical tuberculomas (Table 4 and Figure 2(h)) [38, 41]. In these two cases with tuberculomas, after conversion to negative results of WR-QNRT-PCR assay, transient positivity was once again revealed without any symptoms of meningitis during the course of ATT [38, 41]. In general, tuberculomas often occur along with TBM, but certainly may occur in the absence of TBM [2]. Tuberculomas in these two cases disappeared at 5 and 3 months after the initiation of ATT during the follow-up MRI (Table 4) [38, 41]. Interestingly, WR-QNRT-PCR assay results became completely negative along with the disappearance of tuberculomas [38, 41]. The authors speculate that the transient detections of *M.Tb* DNA by WR-QNRT-PCR assays in these two cases during the ATT were correlated with tuberculomas. A small amount of *M.Tb* might have survived within the tuberculomas. The high sensitivity of WR-QNRT-PCR assay might have detected a little DNA of extinct *M.Tb* leaking into CSF along with the rupture of tuberculomas by ATT. These findings suggest that the combination of molecular-based techniques and neuroradiographic techniques is a promising and powerful diagnostic tool for TBM and tuberculomas in actual clinical practice. To our knowledge, there have been no previous reports on cases such as these two cases; therefore, they may be regarded as clinically important. Moreover, in simple regression analysis, significant correlation (*R* ^2^ = 0.597, *P < 0.0001) was demonstrated between *M.Tb* DNA copy number and clinical stage of TBM (Figure 2(i)) [41]. These diachronic study results indicate that quantitative analysis by WR-QNRT-PCR assay is very useful for assessing the clinical course of TBM and ATT response [41]. To our knowledge, there have been no previous studies that serially assessed the quantity of DNA or bacterial cell numbers of *M.Tb* in CSF samples throughout the clinical course of CNS tuberculosis [41]. In actual clinical application, the WR-QNRT-PCR assay demonstrated significant accuracy and reliability for quantitative detection of *M.Tb* DNA in CSF samples owing to the development of NM-plasmid used as a new internal control. Therefore, this novel assay technique could be regarded as a useful and advanced method for rapidly and accurately diagnosing CNS tuberculosis [40, 41].

However, this novel assay technique is not widely used in routine clinical examination. It may be that the two consecutive amplification steps of WR-QNRT-PCR assay are regarded as a complicated and laborious procedure. Therefore, the authors are developing a further novel and “simpler and low-cost” assay technique with quantification and high-sensitivity almost equivalent to those with the WR-QNRT-PCR assay by one step of standard real-time PCR assay. This developing assay technique is a combination of the whole genome amplification (WGA) method with the real-time (TaqMan) PCR assay technique. The whole genome amplification (WGA) method provides a possibility of amplifying a small amount of high-quality DNA and there are several such techniques using commercially available kits at a reasonable price [50–52]. In particular, in these kits for the WGA method, a kit based on the multiple displacement amplification (MDA) technique has been found in many studies to provide the most balanced genome amplification [50–52]. The MDA technique is a superior method in which genomic DNA is continuously amplified by using Phi29 DNA polymerase of bacteriophage origin and random hexamer primers [50–52]. In this case, the authors used the GenomiPhi V2 kit (GE Healthcare Life Sciences, Uppsala, Sweden) based on the MDA technique. Because a small amount of *M.Tb* DNA extracted and purified from a CSF specimen for use as a template can be amplified directly using the GenomiPhi V2 kit, the first-step PCR in the WR-QNRT-PCR assay can be omitted. This assay technique that is currently being developed can markedly simplify the procedure of WR-QNRT-PCR assay by innovation of the WGA method. Therefore, although it is yet to be reported, its wider use as a beneficial approach for clinical examination is expected in actual clinical practice.

 As described above, CNS tuberculosis including TBM is the severest form of *M.Tb* infection, causing death and serious sequelae [1–5]. In addition, owing to an increasing number of immunocompromised hosts caused by the prevalence of AIDS, increasing numbers of older people, and the wider use of immunosuppressive agents such as corticosteroid, CNS tuberculosis remains a serious clinical and social problem [1–5, 43–46]. In particular, in the underdeveloped and developing countries in Asia and Africa, in which there are many exacerbating social problems such as poverty, overcrowding, and malnutrition, and so forth, the epidemic of *M.Tb* infection including TBM is regarded as a serious social and demographic crisis [1, 2, 43–46]. The authors consider that the novel assay techniques that we have developed are needed and should be used in these places. The wider use of our novel assay techniques will lead to an increase of the number of definitively diagnosed cases within the early stage of TBM and the improvement of treatment results for TBM; therefore, they should make a significant medical and social contribution in these countries. It is hoped that our efforts to develop novel and more rapid, accurate, highly sensitive, quantitative, simple, and low-cost assay techniques for the diagnosis of CNS tuberculosis will help to improve the level of medical care globally, particularly in underdeveloped or developing countries.

## 8. Conclusion

Recently, instead of the conventional “gold standard” based on bacteriological techniques, various approaches have been attempted for rapid and accurate diagnosis of CNS tuberculosis with high sensitivity. In this paper, an overview of recent progress of the NAA methods, mainly highlighting the PCR assay technique, was presented. In particular, the innovation of nested PCR assay technique is worthy of note given its contribution to improve the diagnosis of CNS tuberculosis. Although a novel assay technique, which is internally controlled and combines the high sensitivity of nested PCR with the accurate quantification of real-time (TaqMan) PCR, namely, “WR-QNRT-PCR assay,” is reported as a rapid diagnostic method in TBM, it is not widely used in routine clinical examination. This novel assay technique with high sensitivity in addition to accurate quantification is useful for not only the rapid diagnosis of CNS tuberculosis but also the prediction of prognosis and assessing the effect of ATT during the clinical course of TBM. Therefore, in actual clinical practice, its wider use for the diagnosis of CNS tuberculosis is expected in the future.

## Figures and Tables

**Table 1:** Performance of PCR-based assays for the diagnosing TBM.

Author	Reported year	Assay technique	Specimens and cases	% Sensitivity	% Specificity	Reference
		Commercially available NAA assays				
Bonington et al.	1998	Roche amplicor PCR	83 CSF/69 patients (40 TBM, 29 non-TBM): South Africa	60	100	[6]
Lang et al.	1998	Modified Gen-Probe MTD	84 CSF and children (24 TBM, 60 non-TBM): Dominica	83	100	[7]
Bonington et al.	2000	Roche Cobas Amplicor PCR	83 CSF/69 patients (40 TBM, 29 non-TBM): South Africa	17.5	100	[8]
Chedore and Jamieson	2002	Gen-Frobe MTD	311 CSF: Canada	^†^93.8	^†^99.3	[9]
Pai et al.	2003	Review and Meta-Analysis	14 studies with commercial NAA assays	56	98	[10]
Thwaites et al.	2004	Gen-Probe MTD	341 CSF/152 patients (73 TBM, 79 non TBM): Vietnam	38	99	[11]
Cloud et a1.	2004	Modified Gen-Probe MTD	27 CSF specimens spiked with M. *tuberculosis *	17–100	100	[12]

		Other PCR-based assays				
Kaneko et al.	1990	MPT64 single PCR	26 CSF and patients (6 TBM, 20 non-TBM): Japan	83.3	100	[13]
Shanker et al.	1991	MFT64 single PCR	85 CSF and patients (34 TBM, 51 non-TBM): India	65	88	[14]
Donald et a1.	1993	IS6110 single PCR	43 CSF/20 TBM chidren: South Africa	80	—	[15]
Lee et al.	1994	IS6110/65kDa antigen/MFT64 Single FCR	27 CSF and patients (6 TBM, 21 non-TBM): Singapore	100/83/83	38/67/90	[16]
Liu et al.	1994	MFT64 nested PCR	100 CSF and patients (21 TBM, 79 non-TBM): Taiwan	90	100	[17]
Folgueira et al.	1994	IS6110 single PCR	25 AIDS patients (11 TBM, 14 non-TBM): Spain	82	100	[18]
Scarpellini et al.	1995	IS6110 nested PCR	68 CSF/36 AIDS patients (12 TBM, 24 non-TBM): Italy	100	100	[19]
Lin et al.	1995	MFT64 single PCR	47 CSF/45 patients (18 TBM, 27 non-TBM): Taiwan	70	100	[20]
Kox et al.	1995	IS6110 single PCR	42 patients (24 TBM, 18 non-TBM): The Netherlands	48	100	[21]
Nguyen, et a1.	1996	IS6110 single PCR	136 TBM patients: Vietnam	32	100	[22]
Seth et a1.	1996	MFT64 single PCR	89 CSF and patients (40 TBM, 49 non-TBM): India	85	94	[23]
Wei et al.	1999	IS6110/65kDa antigen/MTF40 multiplex nested PCR	11 CSF and patients (5 TBM, 6 non-TBM): China	60	66	[24]
Caws et al.	2000	IS6110 nested PCR	131 TBM patients: United Kingdom	^†^75	^†^94	[25]
Martins et al.	2000	MFT64 nested FCR	73 specimens (30 PF, 26 PB, 17 CSF): Brazil	^†^70	^†^88	[26]
Brienza et al.	2001	MPT64 nested PCR	91 patients (41 TBM, 50 non-TBM): Brazil	53	100	[27]
Narayanan et al.	2001	IS6110/TRC4 single PCR	96 CSF and patients (67 TBM, 29 non-TBM): India	80.5/91	79/76	[28]
Desai et al.	2002	pKSIO single PCR	120 CSF and patients (105 TBM, 15 non-TBM): India	31	100	[29]
Rafi and Naghily	2003	Single PCR (target not available)	36 CSF and patients (29 TBM, 6 non-TBM): Iran	86.2	100	[30]
Kulkarni et a1.	2005	38 kDa protein single PCR	60 CSF and patients (30 TBM, 30 non-TBM): India	90	100	[31]
Takahashi et al.	2005	MPT64 nested PCR	29 CSF and patients (9 TBM, 20 non-TBM): Japan	100	100	[32]
Takahashi and Nakayama	2006	MPT64 QNRT-PCR	29 CSF and patients (9 TBM, 20 non-TBM): Japan	100	100	[33]
Quan et a1.	2006	IS61 10 single PCR	74 CSF and patients (25 TBM, 49 non-TBM): China	75	93.7	[34]
Desai et al.	2006	Single PCR (target not available)	57 CSF and patients (30 TBM, 27 non-TBM): India	66.7	100	[35]
Rafi et al.	2007	IS6110 single PCR, MPT64/65kDa antigen nested PCR	176 CSF and patients (75 TBM, 101 non-TBM): India	98/91/51	100/91/92	[36]
Rafi and Naghilys	2007	IS6110 uniplex (single) PCR	945 CSF and patients (677 TBM, 268 non-TBM): India	76.4	89.2	[37]
Takahashi et al.	2007	MPT64 QNRT-PCR	63 CSF/28 patients (8 TBM, 20 non-TBM): Japan	55.8	100	[38]
Deshpande et a1.	2007	IS6110 Single PCR	80 CSF and patients (51 TBM, 29 non-TBM): India	91.4	75.9	[39]
Takahashi et al.	2008	MPT64 WR-QNRT-PCR	96 CSF/53 patients (24 TBM, 29 non-TBM): Japan	95.8	100	[40, 41]
Haldar et al.	2009	devR qRT-PCR	167 CSF and patients (81 TBM, 86 non-TBM): India	87.6	92	[42]

NAA: nucleic acid amplification, CSF: cerebrospinal fluid, PF: pleural fluids, PB: pleural biopsies, QNRT-PCR: quantitative nested real-time PCR,WR-QNRT-PCR: wide range quantitative nested real-time PCR, pRT-PCR: quantitative real-time PCR, ^†^: results versus culture as gold standard.

**Table 2:** Sequences of primers and TaqMan probes for WR-QNRT-PCR assays.

Objective	Target	Type	Sequence	PCR product size
First step PCR	Wild *M.Tb *DNA (MPT64) or W-plasmid	WFl: Outer wild forward primer WRl: Outer wild reverse primer	5′-ATCCGCTGCCAGTCGTCTTCC-3′ Total 21 nucleotides, A:2, T:6, G:4, C:9 (GC% 62) 5′-CTCGCGAGTCT AGGCCAGCAT-3′ Total 21 nucleotides, A:4, T:4, G:6, C:7 (GC% 62)	239 bp
	New internal control (NM-plasmid)	MFl: Outer mutation forward primer MRl: Outer mutation reverse primer	5′-TCGATTCTGTCCCACCGCCGT-3′ Total 21 nucleotides, A:2, T:6, G:4, C:9 (GC% 62) 5′-AGACTCGACGCGTAGTCCTCG-3′ Total 21 nucleotides, A:4, T:4, G:6, C:7 (GC% 62)	

	Wild *M.Tb *DNA (MPT64) or W-plasmid	TqMn-WF2: TaqMan inner wild forward primer TqMn-WR2: TaqMan inner wild reverse primer	5′-GTGAACTGAGCAAGCAGACCG-3′ Total 21 nucleotides, A:7, T:2, G:7, C:5 (GC% 57) 5′-GTTCTGATAATTCACCGGGTCC-3′ Total 22 nucleotides, A:4, T:7, G:5, C:6 (GC% 50)	77 bp
Second step PCR	New internal control (NM-plasmid)	TqMn-MF2: TaqMan inner mutation forward primer TqMn-MR2: TaqMan inner mutation reverse primer	5′-AGATCGGATAGCCAGCACGGA-3′ Total 21 nucleotides, A:7, T:2, G:7, C:5 (GC% 57) 5′-TGCGCTGCGTCGACATATTCT A-3′ Total 22 nucleotides, A:4, T:7, G:5, C:6 (GC% 50)	
	Wild* M. Tb *DNA (MPT64) or W-plasmid	TqMn-W-VIC: TaqMan probe-wild-VIC	5′-VIC-TATCGAT AGCGCCGAATGCCGG-TAMRA-3′ Total 22 nucleotides, A:5, T:4, G:7, C:6 (GC% 59)	
	New internal Control (NM-plasmid)	TqMn-M-FAM: TaqMan probe- mutation-FAM	5′-FAM-ATGGGACGGCTAGCAATCCGTC-TAMRA-3′ Total 22 nucleotides, A:5, T:4, G:7, C:6 (GC% 59)	

Underline: artificial sequence.

**Table 3:** The summary of basal clinical features in 24 patients with suspected TBM.

Patient No.	Age/Sex	*Clinical stage	Basal CSF findings (before treatment)						Single PCR assay	Nested PCR assay	WR-QNRT-PCR assay) (copies/mL CSF)	Cranial MRI findings	*M.Tb outside *CNS	Period up to initial sample collection	ATT responsE
			Cells (/*μ*L)	Protein (mg/dL)	Glucose (mg/dL)	ADA (IU/L)	AFB smear	*M.Tb *culture							
Confirmed cases															

1	73/M	III	288	299	13	23.4	−	+	+	+	28721	ME, HC, CVD, IFM	Sp, GA	About 3 weeks	Noneffective
2	76/M	III	165	569	46	12.3	−	+	+	+	10028	ME, CVD	Sp	2 days	Effective
3	28/M	III	605	434	25	16.3	−	+	−	+	22571	ME, HC, CVD, IFM	Sp	About 1 month	Noneffective
4	38/M	II	76	637	18	6.5	−	+	−	+	7161	HC, IFM	Sp, GA	1 day	Effective
5	53/F	III	344	354	38	10.3	−	+	+	+	4547	IFM	−	1 day	Effective
6	72/F	III	247	329	57	18.4	−	+	−	+	6340	HC, IFM	−	7 days	Noneffective
7	34/M	II	612	320	18	20.2	−	+	+	+	1243	−	−	Not available	Effective
8	42/M	II	418	456	36	22.6	−	+	+	+	10532	ME, IFM	Sp	About 2 weeks	Effective

Highly probable cases															

9	35/F	II	208	300	13	16.3	−	−	−	+	7892	ME, HC, CVD, IFM	−	7 days	Effective
10	65/F	I	107	70	48	7.8	−	−	−	+	1904	−	−	3 days	Effective
II	52/M	II	18	135	54	8.6	−	−	−	+	5858	ME, HC, CVD, IFM	Sp, GA	1 day	Effective
12	24/F	I	30	25	30	4.4	−	−	−	+	5436	IFM	−	1 day	Effective
13	44/F	III	60	70	52	N.D.	−	−	−	+	9600	CVD	−	1 day	Effective
14	59/F	II	40	359	78	3.7	−	−	−	+	5112	HC	−	About 1 month	Effective
15	44/M	III	117	87	48	3.9	−	−	−	+	8400	ME	Sp, GA	About 3 weeks	Noneffective
16	40/M	III	800	188	66	12	−	−	−	+	7050	CVD	−	1 day	Effective
17	30/F	III	720	211	50	9.7	−	−	−	+	5596	IFM	−	5 days	Effective
18	20/F	II	442	164	46	17.6	−	−	−	−	Not detected	IFM	GA	Not available	Effective
19	63/M	III	75	84	47	15.9	−	−	−	−	76	ME	Sp	Not available	Effective
20	63/F	II	34	294	30	12.7	−	−	−	+	188	HC	−	Not available	Effective
21	53/M	III	76	81	82	16.9	−	−	−	+	2592	ME, IFM	Sp	1 day	Effective
22	51/M	III	227	155	34	12.7	−	−	−	+	636	ME, CVD, IFM	−	1 day	Effective
23	66/M	III	129	120	58	4.7	−	−	−	+	1600	ME	−	4 day	Effective
24	2/F	II	193	119	30	8.3	−	−	−	+	1444	ME, HC	−	Not available	Effective

(a) The clinical criteria suggestive for TBM are fever, headache, and neck stiffness of more than 1-week duration.

(b) supporting evidence for TBM include (1) compatible abnormal CSF findings that included increased white cell counts with lymphocytes predominating, c -hypoglycorrhachia, protein concentration >100 mg/dL, adenosine deaminase (ADA) ≧10 IU/L and negative results for routine bacterial and fungal cultures; (2) magnetic resonance imaging (MRI) findings suggesting tuberculous involvement of the CNS (basal exudates, hydrocephalus and intracranial focal mass, etc.); (3) presence of tuberculosis in the body outside of the CNS or a history of tuberculosis; (4) clinical response to antituberculosis therapy.

The suspected TBM cases were classified as “confirmed” cases (having the bacterial isolation for *M.Tb *such as CSF culture positive) or “highly probable” cases (meeting all the above clinical criteria and with all three supporting evidences positive).

*According to the clinical stages defined by the British Medical Research Council: stage 0: no definite neurologic symptoms, stage I: slight signs of meningeal irritation with slight clouding of consciousness, stage II: moderate signs of meningeal irritation with moderate disturbance of consciousness and neurologic defects, stage III: severe disturbance of consciousness and neurologic defects.

CSF: cerebrospinal fluid, PCR: polymerase chain reaction, TBM: tuberculous meningitis, *M.Tb*:* Mycobacterium tuberculosis*, ADA: adenosine deaminase, CNS: central nervous system, ME: meningeal enhancement, HC: hydrocephalus, CVD: cerebrovascular disorder, IFM: intracranial focal mass, Sp: sputum, GA: gastric aspirate, ATT: anti-tuberculosis treatment.

**Table 4:** The detail of clinical treatment course of two patients (cases 11 and 12) who had tuberculomas.

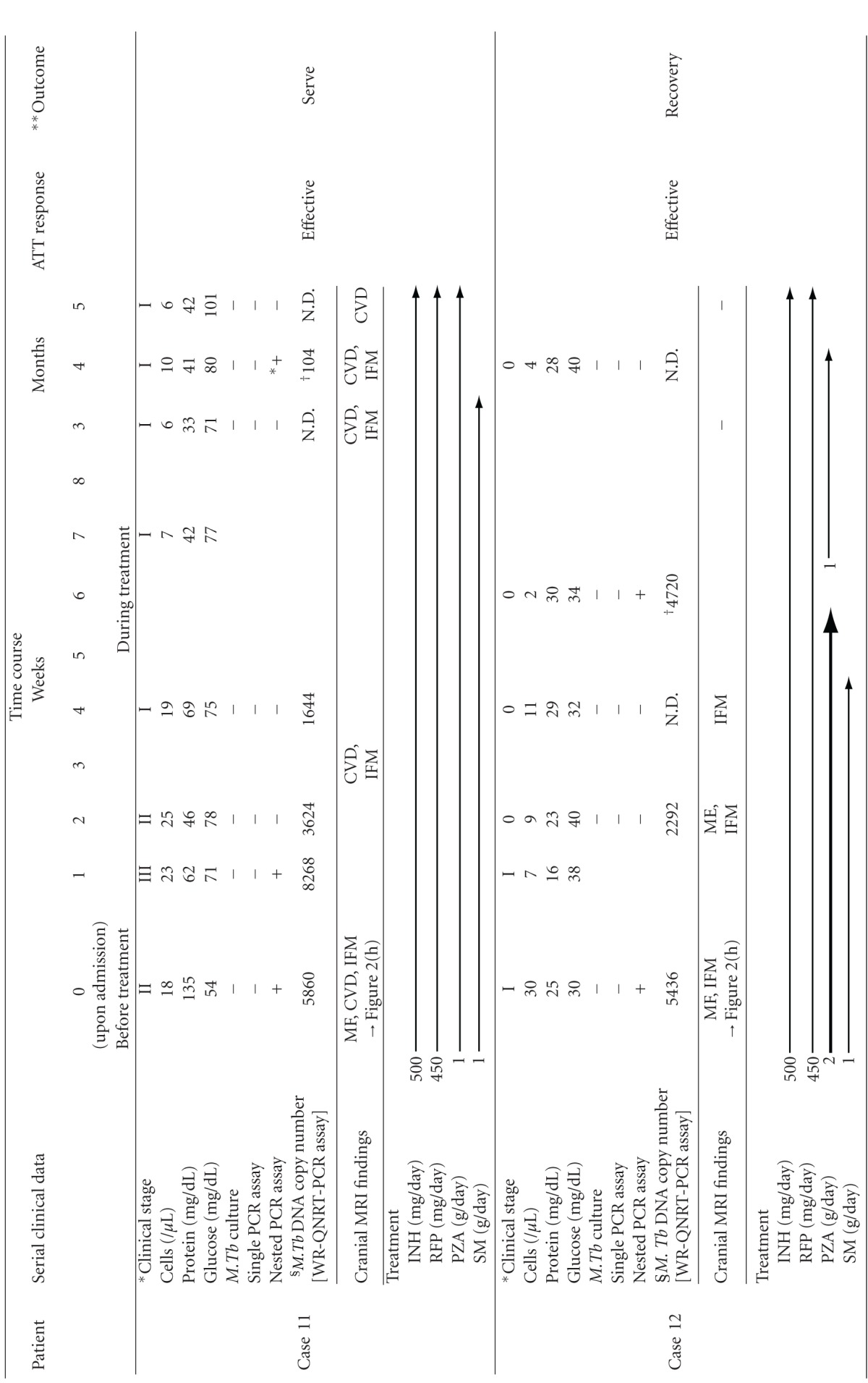

CSF: cerebrospinal fluid, PCR: polymerase chain reaction, *M. Tb*: *Mycobacterium tuberculosis*, ^ +^: positive, ^∗+^: slightly positive, ^ −^: negative, ^§^: per 1 ml CSF, MF: meningeal enhancement, CVD: cerebrovascular disorder, IFM: intracranial focal mass, INH: isoniazid, RFP: rifampicin, PZA: pyrazinamide, SM: streptomycin sulfate; ^†^: transiently positive result.

*According to the clinical stages defined by the British Medical Research Council: stage 0: no define neurologic symptoms, stage I: slight signs of meningeal irritation with slight clouding of consciousness, stage II: moderate signs of meningeal irritation with moderate disturbance of consciousness and neurologic defects, stage III: severe disturbance of consciousness and neurologic defects. **Outcome classified as recovery with minor or no neurologic impairment, severe neurologic impairment, and death.
